# A cross-sectional protocol for experimental tongue high-density surface electromyography to detect and classify radiation-associated hypoglossal neuropathy

**DOI:** 10.1371/journal.pone.0347891

**Published:** 2026-04-29

**Authors:** Nathan J. Hansen, Karin Woodman, Christine Peterson, Sheila Buoy, Xiaohui Tang, Shitong Mao, Amy C. Moreno, Stephen Y. Lai, C. David Fuller, Carly E. A. Barbon, Holly McMillan, Nicolaas C. Anderson, Katherine A. Hutcheson, Benjamin Sanchez, On behalf of the OPC-SURVIVOR Program Working Group

**Affiliations:** 1 Department of Electrical and Computer Engineering, The University of Utah, Salt Lake City, Utah, United States of America; 2 Department of Neurology, Vanderbilt University, Nashville, Tennessee, United States of America; 3 Department of Biostatics, Division of Discovery Science, The University of Texas, MD Anderson Cancer Center, Houston, Texas, United States of America; 4 Department of Head and Neck Surgery, Division of Surgery, The University of Texas, MD Anderson Cancer Center, Houston, Texas, United States of America; 5 Division of Radiation Oncology, The University of Texas, MD Anderson Cancer Center, Houston, Texas, United States of America; 6 Department of Neurology, Baylor College of Medicine, Houston, Texas, United States of America; 7 Department of Electrical and Computer Engineering, The University of Illinois Chicago, Chicago, Illinois, United States of America; 8 Richard and Loan Hill Department of Biomedical Engineering, The University of Illinois Chicago, Chicago, Illinois, United States of America; All India Institute of Medical Sciences, INDIA

## Abstract

**Background:**

Hypoglossal neuropathy is the most common lower cranial neuropathy detected as a delayed sequelae of Human Papillomavirus (HPV) -driven oropharyngeal cancer (OPC). Needle electromyography (EMG) is the gold standard for electrodiagnostic testing, but it is invasive and relies on subjective interpretation of the EMG signal. This study explores the potential of non-invasive high-density surface electromyography (_HDS_EMG) to detect and quantify hypoglossal neuropathy in OPC survivors.

**Objective:**

In an exploratory study, examine the feasibility of _HDS_EMG for rapid, non-invasive screening of hypoglossal nerve (CN XII) function and estimate the prevalence of hypoglossal neuropathy before and after oropharyngeal radiotherapy, and associate with patient-reported and clinician-graded functional outcomes. Machine learning performance will be measured through sensitivity, specificity, and F1 score, with a target area under the curve > 0.7 based on literature-reported EMG sensitivity and specificity.

**Methods:**

This protocol will recruit patients aged ≥ 18 years who receive radiation therapy for OPC at MD Anderson Cancer Center (MDACC) between 2024–2025 and consent to experimental _HDS_EMG testing. Sanchez Research Lab (The University of Utah, Salt Lake City, UT) will perform data analysis. Clinical data—including electrical impedance measurement (EIM), patient-reported outcomes, dysphagia grading, tongue functions, fibrosis grading, and needle EMG—will be collected from n = 36 patients. Features extracted from _HDS_EMG will be correlated with other clinical outcomes and used to train a machine learning classifier to quantify the severity of hypoglossal neuropathy.

## Introduction

Oropharyngeal squamous cell carcinoma, arising from the base of the tongue and tonsil, is increasing in incidence. We now recognize that many tumors are related to the human papillomavirus (HPV), and this trend is expected to continue without decline until at least 2045, despite current vaccination rates [1–4]. Patients with HPV-attributable oropharyngeal cancer (HPV-OPC) tend to have a more favorable survival prognosis than patients with a history of tobacco use [5].

Furthermore, hundreds of thousands of young survivors of HPV-OPC will live a significant portion of their lives with post-treatment toxicities from radiotherapy [1]. Normal tissue injury from radiotherapy along the hypoglossal nerve (cranial nerve XII), such as radiation-induced fibrosis or lymphedema, causes denervation of the tongue musculature, contributing to swallowing dysfunction and increasing the risk of choking and aspiration pneumonia (airway entry of liquids or food) [6–8]. These symptoms significantly decrease survivors’ quality of life (QOL) and, in the worst case, lead to tracheostomy or feeding tube insertion.

Clinicians identify and assess manifestations of hypoglossal neuropathy using patient-reported outcomes (PRO) questionnaires (e.g., MD Anderson Dysphagia Inventory [MDADI]; MD Anderson Symptom Inventory-Head and Neck [MDASI-HN]) and interviews regarding patient functional status (e.g., Performance Status Scale-Head and Neck Cancer [PSS-HN]). Cross-sectional cohort studies have used these indices to evaluate symptom burden in OPC survivors with and without hypoglossal neuropathy [5,6,9]. These studies found that patients with hypoglossal neuropathy scored 1.54 points worse on the MDASI-HN overall, with the most significant implications on bulbar symptoms, including difficulty with swallowing/chewing, choking, speech/voice, mucus, and fatigue [5]. Furthermore, MDADI, PSS-HN, and PRO scores showed that patients with cranial neuropathy experienced adverse functional status metrics like dietary restrictions, nutritional impairment, and a decline in public food consumption [6]. In that body of studies, the investigators demonstrated substantial risk and burden of lower cranial neuropathy (most commonly CN XII neuropathy) in OPC. However, these insights are subject to limitations of clinical surveillance metrics. The bias inherent in rudimentary clinical detection methods matched to PRO surveys and related measures increases the uncertainty of the conclusions, and symptom reports cannot be expected to mimic physiology or reveal the underlying mechanisms contributing to pathological states.

Current technologies constrain physiological measurement of the tongue. Tongue strength, as manometrically measured by the Iowa Oral Pressure Instrument (IOPI), is shown via meta-analysis as a valid tool for measuring tongue pressure and monitoring tongue treatment progression [10]. Hypoglossal nerve health can be assessed quantitatively using needle electromyography (EMG) of the genioglossus [11]. Experienced neurologists detect neuropathy by grading electromyograms on a numeric grading system from 0 (normal) to 4 (spontaneous fibrillation potentials filling the screen) [12,13]. Despite its clear value, needle EMG is invasive, can be performed only by trained specialists, and uses a coarse, subjective clinical grading scale. Action potentials are graded by the prevalence of specific patterns, as determined through visual or auditory analysis. Fibrillation potentials repeat in a definable, regular pattern, typically with a linear increase in the interspike interval. Voluntary MUPs fire in a semi-rhythmic pattern, with interspike interval changes of less than 10% [14].

We propose to investigate tongue high-density surface electromyography (_HDS_EMG) as a non-invasive alternative to needle EMG, motivated by previous work measuring electrical impedance myography with surface electrodes [15–17]. In the published literature, subcranial multichannel _HDS_EMG of the swallowing mechanism has a non-inferior signal-to-noise ratio (SNR) compared to larger gel electrodes [18,19]. Several robust decomposition algorithms exist for selectively reconstructing individual motor unit potentials (MUPs) from the surface [20]. We expect that signal abnormalities indicative of tongue neuropathy will manifest in processed _HDS_EMG signals, as they do in needle EMG, and will correlate with tongue function abnormalities revealed by functional outcomes examination. We propose that a machine-learning approach to _HDS_EMG will reveal the underlying relationship between tongue EMG waveform morphology and the presence of XII neuropathy, allowing for neuropathy grading on a continuous scale. We expect that revealed relationships may enable the early discovery of hypoglossal neuropathy, allowing clinicians to intervene before irreversible progression manifests.

## Materials and methods

### Study design

We propose an exploratory cross-sectional evaluation of hypoglossal nerve function and swallowing among OPC survivors along the cancer care continuum. The project will involve primary data collection of tongue function procedures among an active cohort (the MD Anderson Oropharynx Cohort, “MDA-OPC,” protocol #PA14–0947). Participant recruitment will occur from June 2024 to May 2026. Preliminary results of the primary objective are expected in 2027. PROs, survey responses (PSS-HN, MDASI-HN, MDADI), functional outcomes (IOPI, EIM), EMG recordings, and _HDS_EMG recordings of consenting patients will be stored on the parent cohort study (MDA #PA14–0947). Analyses herein do not overlap with the work of the parent cohort.

### Population

The population will include patients evaluated at the University of Texas MD Anderson Head and Neck Center with a suspected or confirmed pathologic diagnosis of squamous cell carcinoma (SCCA) of the oropharynx, tonsil, or base of the tongue, or of head and neck SCCA of unknown primary. Spanish-speaking participants and pregnant women may be enrolled. In this exploratory analysis, patients will be enrolled regardless of radiation dosage.

### Inclusion criteria

- Patients evaluated at MD Anderson Cancer Center with pathologic diagnosis of SCCA of the oropharynx, tonsil, or base of the tongue, or SCCA of the head and neck of unknown primary who consent to EMG testing.
- If consented post-treatment, there is no evidence of disease

### Exclusion criteria

- Primary surgery for OPC.
- Documented CN XII nerve injury by tumor, surgery, or other source. (Confounds radiation-induced CN XII injury.)
- History of prior head and neck cancer or central nervous system cancer.
- Functionally limiting cardiac, pulmonary, or neuromuscular disease.
- Current tracheostomy.
- History of surgery near the hypoglossal nerve path.
- Platelet count <50,000 cells/mL.
- Patient < 18 years of age.
- Cognitive impairment (unable to complete questionnaires).

### Objectives

#### Primary objective: Examine the feasibility of _HDS_EMG for rapid, non-invasive screening of XII nerve function.

We plan to show that a machine learning algorithm trained on features of _HDS_EMG waveforms collected during a brief series of tongue tasks will detect CN XII neuropathy and grade its severity on a continuous, more sensitive scale than existing EMG. Time-frequency features extracted from _HDS_EMG will be used to detect tongue neuropathy grade using linear discriminant analysis, ensemble machine learning classifiers, and a neural network with a multiclass activation function, providing insight into the relationship between _HDS_EMG and tongue function degradation. Tongue _HDS_EMG will also be decomposed into estimates of constituent MU spike trains for analysis with a convolutional neural network.

#### Secondary objective: Estimate the prevalence of XII neuropathy before and after oropharyngeal radiotherapy and associate it with patient-reported and clinician-graded functional outcomes.

There are no published risk estimates for CN XII neuropathy in OPC using the gold-standard electrophysiologic measurement from EMG. Existing estimates rely on clinical detection, which begins with the patient’s self-reported symptoms and proceeds to physical examination. The patient first notices the clinical manifestation of neuropathy as tongue weakness, often prompting physical examination to identify hallmark features of tongue deviation, atrophy, and fasciculation, which help diagnose CN XII neuropathy. Speech-language pathologists might also use swallow studies, speech samples, and tongue strength measures to track loss of tongue function over time. We expect needle EMG and experimental _HDS_EMG of the tongue in OPC survivors across time to reveal an increasing prevalence of CN XII neuropathy over time, as well as relationships between physiological measures and patient-perceived tongue weakening.

### Data collection

Participants consenting to the tongue testing procedures on MDA protocol #PA14–0947 will participate in measurement at baseline and/or 3–6-, 18–24-, 60-month data collection time points including: a) intramuscular EMG with or without nerve conduction study (NCS), b) tongue surface electrical impedance myography (EIM) and _HDS_EMG, c) a video recording of Lingual range of motion (L-ROM), and d) tongue strength. L-ROM and strength will be assessed using published, validated methods [21,22]. The IOPI digital manometer will measure maximum isometric lingual strength to assess maximum pressures generated by the tongue. Together, the proposed measures will characterize the nature of nerve activity, the composition of the tongue, and its function. Tongue testing procedures require a clinical appointment in the neurology center. They are expected to take about 30 minutes for EMG and up to 30 minutes, including breaks between tasks, for tongue function measures. During the visit, the study team will assign each subject an accession number that corresponds to all their deidentified data at every time point.

### Needle electromyography and nerve conduction

Intramuscular EMG will assess insertional activity in the genioglossus muscle, innervated by CN XII, as a marker of denervation [23]. Needle EMG is reported to have an inter-operator reliability of 0.53 [24]. EMG recordings will be conducted by a cancer neurologist fellowship trained in clinical neurophysiology and denervation potentials graded per standard neurologist’s grading:

0 - None

1 - Persistent, single train of potentials in at least two areas

2 - Moderate number of potentials in three or more areas

3 - Many potentials in all areas

4 - Full interference patterns of potentials

EMG and NCS may also be performed in the trapezius muscle (innvervated by CN XI), as the region is easily accessible for non-invasive testing and represents a muscle within the irradiated field with lower cranial nerve innervation. Both quantitative and qualitative EMG may be assessed [25]. Needle EMG will not be conducted if platelets are < 50,000. The intramuscular EMG will require a clinical visit with the neurologist.

### Tongue electrical impedance myography

This brief, non-invasive procedure will measure electrical conductivity in the tongue as a proxy for tongue composition. The patient’s experience is equivalent to placing a tongue depressor briefly on the tongue. The EIM system comprises a battery-powered recording unit called the user tongue electronic system (UTES) that couples to the user tongue array (UTA) depressor, custom-fabricated by Dr. Sanchez’s lab [15,16,26,27]. A smartphone is paired with the EIM system via Wi-Fi, and a custom software application allows the user to initiate the measurement, display the results, and save de-identified data.

The UTES transmits an imperceptible electrical current during a measurement through the UTA depressor. The voltage generated by the current flowing through the tongue is measured on all remaining surface electrodes. The alternating current frequency ranges from 8 kHz to 256 kHz. Across all frequencies, the current amplitude is limited to 0.1 mA, well within the safety requirements for applied current specified in the International Electrotechnical Commission (IEC) 60601−1 safety standard for medical devices. Primary EIM outcomes will include resistance, reactance, phase, conductivity, and permittivity at selected frequencies.

The UTA depressor will be for single-patient use. Each patient will have a dedicated depressor, which will be air-dried before being stored in an approved, labeled biospecimen bag between time points while not in use. The UTES will be stored with the clinical research staff when not in use and wiped with alcohol before and immediately after use. All data from the device will be automatically de-identified and sent to Dr. Sanchez’s lab for processing and analysis.

The underlying premise is that directional changes in disease tongue EIM signatures will provide new information on tongue health [16,17]. The system results from ongoing research efforts from collaborator Dr. Sanchez and has similar functionality and safety performance to clinical systems for impedance in other body regions. The system has been used to measure the tongue of seven healthy subjects [16]. We have conducted previous work that demonstrates the preliminary feasibility of EIM to distinguish neuropathic and healthy tongues [17]. It is approved as an investigational device for human use under IRB approval at Beth Israel Deaconess Medical Center, Boston, MA (Clinicaltrials.gov Identifier: NCT02118805). The University of Utah has submitted a provisional patent disclosure for tongue technology.

### High-density surface electromyography

_HDS_EMG will be measured similarly to the EIM (as described above using the UTA/UTES system). The recording system will be a commercial TMSi SAGA, 32-channel _HDS_EMG instrument (SAGA32, Twente Medical Systems; Oldensaal, The Netherlands). This investigational device will comply with all FDA regulations under 21 CFR 812. The investigators will comply with monitoring and record keeping, and the device will not be marketed or promoted. The surface electrodes in the tongue depressor will allow us to measure the distribution of electrical activity over a confined tongue area. The electrode grid consists of 32 electroless nickel-gold contacts of 2 mm diameter. The inter-electrode distance is 2.5 mm. The electrode array connects via a clamshell to a commercial _HDS_EMG amplifier, which records signals at a 4 kHz sampling rate. The unipolar channels have RMS noise < 1.5 µV, input impedance > 1 GΩ, and an analog bandwidth of 0–800 Hz.

Each patient will clean and prepare their tongue for the exam by gently wiping it with gauze and drinking a small amount of water. The arrays will be prepped with an alcohol pad and allowed to dry before tasks are performed. The patient will lie supine on a procedure table, and the electromyographer will begin the left tongue relaxation task. The electromyographer (CA) will evaluate needle EMG for spontaneous activity, and then the depressor will be placed on the left tongue at rest for 30 seconds to evaluate spontaneous _HDS_EMG activity simultaneously with needle EMG. For the voluntary MUP potential analysis, the patient will protrude the tongue at approximately 20% of maximum force, and the electromyographer will evaluate EMG during the voluntary contraction. Then the tongue depressor will be placed on the left tongue to record 30 seconds of voluntary muscle activity with EMG and _HDS_EMG simultaneously. The electromyographer will then coach the patient to perform an isometric force ramp, increasing tongue force against the roof of the mouth from zero to maximum over about 10 seconds. These tasks are then repeated in the right tongue. If the patient swallows or speaks during the task, the measurement will be repeated. Table 1 summarizes the total _HDS_EMG data collected. The duration of discharges and the frequency of potentials will be measured at all electrode locations to confirm _HDS_EMG data stability and to reveal tongue fasciculations and recruitment patterns.

**Table 1:** Surface electromyography measurement duration breakdown. Measurements will be recorded in the left tongue and right tongue.

Tongue Side	Measurement	Duration (Seconds)
Left	Rest	30
	Protrusion	30
	Isometric	10
Right	Rest	30
	Protrusion	30
	Isometric	10
	Total	140

Needle EMG and _HDS_EMG procedures will be performed during the same visit. An electromyographer (CA) will evaluate hypoglossal needle EMG submentally to provide a clinical assessment of CN XII health. The patient will then perform the tasks we proposed while the needle is still inserted and _HDS_EMG is collected simultaneously. Finally, the patient will repeat our proposed tasks with only the _HDS_EMG electrodes on the tongue.

### Tongue strength and range of motion

Brief, non-invasive testing will measure tongue function, including strength and range of motion, using published methods. A video recording of L-ROM and strength will be assessed using published, validated methods. The IOPI digital manometer will measure maximum isometric lingual strength to assess the maximum pressures generated by the tongue (inter-operator reliability of 0.75) [28,29].

### Clinician graded lymphedema and fibrosis

Clinician-graded soft tissue palpation sores in the bilateral submental, anterior, and lateral neck will be recorded using the Head and Neck External Lymphedema and Fibrosis (HN-LEF), which has a reported inter-operator reliability of 0.91 [30,31]. This tool uses ordinal scoring to evaluate the type (A-D) and severity grade (1–5) of soft tissue changes.

### Data management and ethics

The data collected for this study are part of a larger cohort effort to collect comprehensive outcomes data from consenting patients in the Oropharynx program at the MD Anderson Cancer Center in Houston, Texas (MDA-OPC). The collection is approved under MD Anderson IRB #PA14–0947 for MD Anderson Oropharynx Cohort patients who consent to tongue testing procedures (optional procedure #2 on the parent consent form). Patients scheduled for appointments in the Head and Neck Center will be identified by either an attending physician or an appropriately trained designee. During clinic appointments, potential subjects will be informed of their eligibility and asked if they would be interested in participating in research. In the privacy of the exam room, physicians or designees with appropriate training will discuss all aspects of the study with potential subjects and answer any questions. Subjects will be given a copy of the Informed Consent and further instructed about the study and the elements of the consent document. The subject will be given as much time as needed to review and discuss the study and consent, and the research nurse, research data coordinator, attending physician, or designee will answer any remaining questions. Non-English-speaking patients will consent to use an MD Anderson Translator and a verbal translation preparatory sheet, in accordance with MD Anderson policies for Informed Consent translation, in that patient’s primary language. Patients may withdraw from the study at any time without any penalty. The MD Anderson Institutional Review Board classifies this study as minimal risk and does not monitor this study for adverse events. The PI is responsible for monitoring adverse events. Risk will be evaluated by the electromyographer upon review of the patient’s medical history and medication.

Since 2015, 1,750 OPC patients have enrolled with detailed, interdisciplinary characterization of exposures, diagnostic/staging, treatment, disease control, and longitudinal collection of validated clinician- and patient-reported survivorship outcomes from diagnosis through 5-year survival. All longitudinal MDA-OPC clinical data is securely stored in REDCap® and DMI, an institutionally secured Data Management Services platform. Our DMI-based MDA-OPC database contains 14 data forms, 1182 imported variables, 439 manually entered variables, and 18 customized reports, with nightly updates from integrated data sources. Anyone interested in analyzing the collected data can submit a request to the database oversight committee (oropharynxprogrampro@mdanderson.org). Functional outcomes, PROs, and electrodiagnostic data will be de-identified and provided to the Sanchez Research Lab for analysis under MD Anderson IRB #PA14–0947. Code and models will be stored in a public GitHub repository within the group “SanchezResearchLab/hdsemg_pipeline.” Because the software is not complete, the source code will be made publicly available when the analysis is complete.

### Outcomes definition

#### Primary objective.

For analysis of the primary objective, the primary endpoint will be _HDS_EMG classification of XII neuropathy (yes/no, then graded) in relation to the gold-standard needle EMG classification. We expect to show that features such as frequency of interference patterns and tongue fasciculations correlate between _HDS_EMG and needle EMG, demonstrating that _HDS_EMG is a feasible, rapid, non-invasive biomarker for tongue neuropathy. We will consider _HDS_EMG feasible for this exploratory analysis if more than 80% of the signal recordings meet the quality criteria. We also expect that a neural network with a multiclass activation function will grade tongue neuropathy progression on a continuous scale.

#### Secondary objective.

For analysis of the secondary objective, the primary endpoint of XII neuropathy will be grade>0, classification of denervation potentials per needle EMG. Secondary endpoints of tongue function will include the total L-ROM score and MILS in the anterior and posterior tongue.

### Data analysis and machine learning plan

**Fig 1:**
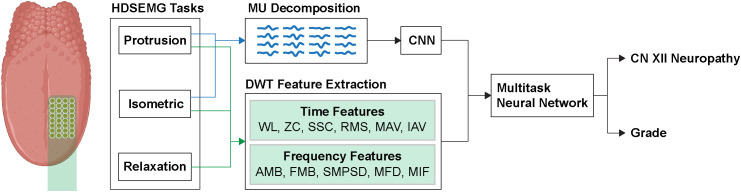
Tongue _HDS_EMG data processing pipeline. Tongue _HDS_EMG recordings from three patient tasks will be decomposed via DWT, and time-frequency features will be selected using linear discriminant analysis. Voluntary contraction tasks will be decomposed into motor unit spike train estimates using MUEdit, and a CNN will be trained on the resulting MU estimates. A final multitask neural network will make a final CN XII neuropathy prediction from all the data. Abbreviations: _HDS_EMG, High-density surface electromyography; MU, Motor unit; CNN, Convolutional neural network; WL, Waveform length; ZC, Zero crossings; SSC, Sign slope changes; RMS, Root-mean-square amplitude; MAV, Mean absolute value; IAV, Instantaneous absolute value; AMB, Amplitude-modulated bandwidth; FMB, Frequency-modulated bandwidth; SMPSD, Spectral moment of power spectral density; MFD, Mean first derivative of instantaneous frequency; MIF, mean instantaneous frequency; CN XII, Cranial nerve XII.

Clinical measures will be used to confirm the diagnosis and grade of neuropathy. We expect a pilot sample size of 36 patients with needle EMG and _HDS_EMG to provide ample statistical power to develop a machine-learning classification algorithm, since acquiring 2 minutes and 20 seconds of EMG data at 10 kHz will yield 50.4 million values (36 pts x 140 sec x 10 kHz). For 32-channel _HDS_EMG at 4 kHz (the threshold for the SAGA device), that is 645.12 million values (36 patients x 140 sec x 4 kHz x 32 EMG simultaneous channels). Still, we acknowledge our limitations in determining the optimal sample sizes, as we do not yet have _HDS_EMG data in OPC patients. If the initial analysis shows insufficient performance, we will assess EMG and _HDS_EMG subsets to further increase the training and test dataset sizes. Fig 1 depicts the general data processing pipeline that will translate _HDS_EMG measures into CN XII neuropathy grade. Signals will be preprocessed using MATLAB R2023a software (MathWorks; Natick, MA). Experimental _HDS_EMG will be denoised using a bandpass filter from 20–500 Hz. The signal quality assessment for each task consists of the following. First, a rolling mean absolute value of each channel (window size 400 samples) will represent the apparent effort exerted by the patient.

- Relaxation: None are rejected because motion artifacts appear similar to fasciculations on _HDS_EMG.
- Protrusion: Protrusion task channels will be rejected if the range of the apparent effort curve is larger than 2 standard deviations of the signal mean absolute value.
- Isometric: Isometric task channels will be rejected if the Pearson’s Correlation of the apparent effort curve shows a non-positive slope or a positive slope with r-squared < 0.2.

All accepted signals are divided into 2-second segments for feature extraction. The Discrete Wavelet Transform (DWT) will be used to extract time series features (waveform length, zero crossings, slope signal changes, root mean square, autoregressive coefficient, mean absolute value, and integrated mean absolute value) and time-frequency features (mean instantaneous frequency, mean first derivative, amplitude-modulated bandwidth, frequency-modulated bandwidth, and spectral moment of power spectral density) [32]. We will perform Mann—Whitney U-tests with α = 0.05 to determine whether these features are relevant to neuropathy through the following comparisons: reduced recruitment vs. no reduced recruitment; polyphasia vs. no polyphasia; increased amplitude vs. no increased amplitude; and increased duration vs. no increased duration. To further demonstrate the clinical relevance of _HDS_EMG features, we will use Pearson’s Correlation Coefficients and multiple regression analysis to express correlations between _HDS_EMG features and tongue functional outcomes (L-ROM and IPOI pressure).

The full dataset will be partitioned patient-wise into 70% for training, 20% for development, and 10% for validation. Features from the training set that discriminate neuropathy severity will be selected using linear discriminant analysis. Missing data may occur during time-frequency feature extraction, as some calculations can trigger divide-by-zero errors, especially for low-frequency components. If this occurs, such examples will be rejected as artifacts. If any class label is represented by less than 10% of the data, recording-wise mean imputation will be performed to augment the number of unique examples in the underrepresented classes.

We intend to train an initial decision tree and support vector machine (SVM), followed by ensemble methods using both trees and SVMs, where possible: Random Forest Classifier, Gradient Boost, and Bagging. We will also train a neural network classifier with a softmax activation function for multiclass classification. Hyperparameter optimization will be performed using 5-fold cross-validation and verified using the development set. To account for potential class imbalance, all ensembled learning methods will be trained using class-weighted sampling to ensure that individual classifiers within the ensemble see class-balanced subsets of the data. Machine learning will be performed using SciKit Learn [33].

The 32-channel _HDS_EMG signals are a convolutive mixture on an _HDS_EMG recording and can be extracted using convolutive blind source separation (BSS). Statistical motor unit decomposition software (MUEdit, Avrillon et al.) [34] will be used to extract estimated spike trains from each suite of voluntary contraction _HDS_EMG tasks. Spike train estimates include valuable MU features such as the number of MUs and discharge rate. We plan to construct shimmer plots for each of the 32 spatial recordings by aligning the identified MU estimate peaks [34]. These aligned MU examples will train a convolutional neural network (CNN), using MU peak morphology to predict neuropathy grade. The CNN will be built with PyTorch and trained with help from Optuna for hyperparameter optimization [35,36].

For the secondary objective, we will estimate the prevalence of neuropathy at three time points (pre-treatment, 3–6 months after treatment, and 18 months to 5 years after treatment), along with exact 95% binomial confidence intervals (CIs). Logistic regression models will be fit with XII neuropathy as a binary response variable adjusted for sampling weights and clinical covariates. For hypothesis 1.1, we will model time as a binary variable (0 = pre-RT and 1 = post-RT), pooling the two post-RT groups (n = 24) versus the pre-RT group (n = 12). Time will be included as a continuous variable (i.e., months pre- or post-RT). We expect an exploratory sample of 36 patients who consent to tongue testing, with equal allocation across 12 patients in one of three groups based on the timing of EMG collection relative to their cancer treatment: pre-treatment, early post-treatment, and late post-treatment. Twelve patients per group ensure a maximum half-width of the 95% CI of 0.29. After treatment, we expect the prevalence of neuropathy to be approximately 40%, based on our published data that 10% of survivors develop clinically detectable neuropathy [9,37]. Unpublished data from the NIH intramural laryngeal study section found that 90% of chronic radiation-associated dysphagia cases have EMG-detectable denervation [38]. The investigators’ data show that 100% of patients with DIGEST grade ≥2 dysphagia who are ≥ 2 years disease-free HNC survivors have EMG detectable neuropathy as early as 2 years post-RT (38% of whom have subclinical neuropathy). Logistic regression on a binary independent variable (pre- vs. post-RT) with a sample size of 36 (n = 12 pre- and n = 24 post-RT) achieves 80% power (α = 0.10) to detect a difference in the probability of neuropathy from 5% pre-RT to 42% post-RT.

### Evaluation criteria

The performance of machine learning classifiers will be divided into two classification settings: binary (CN XII neuropathy vs. no confirmed CN XII neuropathy) and multiclass (neuropathy grade). In binary classifiers, we aim for AUC ≥ 0.70 to support _HDS_EMG as an inexpensive, non-invasive quantitative metric for screening OPC survivors for CN XII neuropathy. Narayanaswami et al. report that electromyographers with 10–20 years of experience have a diagnostic sensitivity of 0.77 and a specificity of 0.71 for neuropathy. [24] This will serve as our benchmark for reliable performance for all binary model development. For the 10% validation set, we will measure model performance simply by the percentage of correctly diagnosed cases of neuropathy. As a secondary validation outcome, we will compare the _HDS_EMG neuropathy severity grade with the gold-standard needle EMG grades.

Multiclass classification will use micro- and macro-F1 scores to evaluate the validity of a refined CN XII neuropathy grade prediction. Saliency mapping for the CNN will reveal which signal regions contribute to each neuropathy grade prediction. Machine learning models will be evaluated using SHapley Additive exPlanations (SHAP) to investigate which features contribute to the model predictions [39].

### Patient and public involvement

Patients and the public were not involved in the design. However, the investigators have recently engaged patient research partners for a program of research on delayed oropharyngeal cancer sequelae and expect to involve participants in the implementation and dissemination of the research plan, including consultation on recruitment/data collection strategies and on integrating these technologies into clinical trials.

### Dissemination strategy

Research data collection began in June 2024. Data collection will continue through May 2027, and we are targeting December 2027 for completion of data analysis. We will perform preliminary analyses and report progress in the annual RPPRs associated with this study’s NIH funding. We expect to submit our complete findings to a suitable journal by March 2028. We will disseminate the findings of our analysis to the scientific and medical community through publication in a peer-reviewed journal and presentation at relevant conferences for head and neck surgery, electrodiagnostics, biomedical engineering, and machine learning.

## Discussion

This study will primarily investigate the feasibility of tongue _HDS_EMG to monitor CN XII neuropathy in OPC survivors. Clinical gold-standard functional and neurophysiological data will be collected alongside experimental data to provide a diverse set of correlatives that strengthen the clinical relevance of the experimental findings. By synchronizing gold-standard needle EMG, _HDS_EMG, and machine learning methods for time series signals, our analysis will elucidate relationships between morphology and the continuum of nerve health and function in OPC survivors. Comparisons between electrodiagnostic methods, functional outcomes, and patient-reported outcomes will reveal links between underlying physiology and tongue functional degradation. Oropharyngeal cancer survivors’ outcome data will be collected into a central database, with access available upon request, facilitating research collaboration.

Our long-term goal is to provide a simple quantitative metric to detect loss of neurophysiologic signal in the tongue, thereby stratifying those at risk for profound late-effect syndromes earlier in their survivorship. Should this _HDS_EMG neurophysiologic marker prove possible, it may allow doctors to employ simple pharmacologic solutions such as early pulsed, high-dose steroids (e.g., similar to the Bell’s palsy model or use in radiation-induced optic nerve injury) [40,41] that depend on early detection to avoid the functional catastrophe of subsequent muscle atrophy and aberrant reinnervation.

The collected data may fail to demonstrate that _HDS_EMG feature machine learning classification adequately detects XII neuropathy, in which case, concurrent EIM offers a compelling alternate non-invasive biomarker for denervation-mediated compositional changes that we will also collect in this protocol [16,17].

Fundamentally, if the data fail to show that _HDS_EMG features can be used to train machine learning classifiers to screen for XII subclinical neuropathy, the comprehensive follow-up of the cohort remains compelling. That is, linking the EMG denervation classification with functional outcomes (e.g., IOPI, L-ROM, and PROs) alongside non-invasive EIM as a physical compositional tongue measure will allow estimation of prevalence, functional translation, as well as comprehensive hypothesis-generating analyses of post-RT lingual denervation in a manner explored for other nerves but not yet quantified for radiation-associated CN XII [42].

This protocol seeks to provide technical solutions and mechanistic insight to refine management of just one domain of HNC survivorship (neural-mediated radiation-associated dysphagia). However, the work must be taken in the broader context of the rapidly evolving landscape of HNC, where progress in tumor microenvironment, immunotherapy, optimized radiation, and targeted therapy promise to transform care models. Comprehensive, mechanistically informed multidimensional toxicity assessments will be one of many advances the investigators hope to realize in the next generation of head and neck cancer care. For example, we recognize the role of the CXCL12-CXCR4 signaling axis in cancer metastasis and the utility of nanomedicine strategies to enhance immunogenic cell death to improve immunotherapy efficacy [43–45]. If successful, methods for non-invasive hypoglossal nerve monitoring will need to be integrated with similar emerging multidimensional protocols for other toxicity states, such as radiation-induced ocular complications and mental health care during cancer treatment [46–48].

We acknowledge that only exploratory interpretations can be made from models trained on patients at a single site and evaluated by a single physician. Additionally, several other factors, such as primary tumor site, radiation dose, chemotherapy, and time since radiation treatment, are not controlled for in this study. We intend to investigate only the diagnostic utility of _HDS_EMG and EIM, but we cannot comment directly on factors that contribute to the risk of CN XII neuropathy. Future work may include a follow-up study stratifying patients by radiation dose to control for its influence on LCNP risk. The effects of radiation and chemotherapy on neuropathy incidence have also been studied within our group and may be replicated with respect to _HDS_EMG. [48,49] The generalizability of our work will be limited by our exploratory sample size of N = 36. Future work would involve patients at multiple sites and use a group of physicians to evaluate needle EMG. Additionally, _HDS_EMG analysts would be blinded to the labeled diagnosis of a multi-site test set of patients to further validate generalizability. Our work will inform future sample size estimates by establishing benchmark sensitivity and specificity values for neuropathy classification using _HDS_EMG.

## Conclusion

We hypothesize that tongue _HDS_EMG analysis, paired with time-frequency feature extraction, MU decomposition, and machine learning, will be feasible for monitoring CN XII health in OPC survivors at risk for CN XII neuropathy. Such a tool would provide a non-invasive means for quantitative diagnosis and monitoring of CN XII neuropathy.

## Supporting information

S1 File English informed consent and protocol. This is the parent consent form for #PA14–0947. See pages 4–5 for the optional procedures #2 consent.
(PDF)

S2 File Spanish informed consent and protocol. This is the Spanish parent consent form for #PA14–0947. See pages 4–6 for the optional procedures #2 consent.
(PDF)
