# Supplementary material for: A cross-sectional protocol for experimental tongue high-density surface electromyography to detect and classify radiation-associated hypoglossal neuropathy
Source: PLoS One. 2026 Apr 29;21(4):e0347891. doi: 10.1371/journal.pone.0347891 (PMC13127915; doi:10.1371/journal.pone.0347891)
Supplement: S1 File — This is the parent consent form for #PA14–0947. See pages 4–5 for the optional procedures #2 consent. (PDF) [file pone.0347891.s001.pdf]

THE UNIVERSITY OF TEXAS
MD Anderson Cancer Center

Informed Consent

INFORMED CONSENT/AUTHORIZATION FOR PARTICIPATION IN RESEARCH WITH OPTIONAL PROCEDURES

Oropharynx Program Database
PA14-0947

Study Chair: Amy Moreno, MD

____________________________ ________________________
Participant's Name Medical Record Number

This is an informed consent and authorization form for a research study. It includes a summary about the study. A more detailed description of procedures and risks is provided after the summary.

This research has been reviewed and approved by an Institutional Review Board (IRB - a committee that reviews research studies).

STUDY SUMMARY

The goal of this research study is to collect information and leftover tumor tissue samples from patients with cancer of the head and neck, including the oropharynx, tonsils, and base of the tongue. Researchers will use these samples and information to create a database and research bank for use in future research related to cancer.

This is an investigational study.

Future patients may benefit from what is learned from this study. There are no benefits for you in this study.

Your participation is completely voluntary. Before choosing to take part in this study, you should discuss with the study team any concerns you may have, including potential expenses and time commitment.

You can read a list of potential risks below in the Possible Risks section of this consent.

There will be no cost to you for taking part in this study.

You may choose not to take part in this study.

1. STUDY DETAILS

Up to 5000 participants will be enrolled in this study. All will take part at MD Anderson.

If you agree to take part in this study:

- The study staff will collect information about the disease and your treatment history from your medical record.
- You may complete up to 4 questionnaires about your quality of life and side effects from treatment before your regularly scheduled treatment, during treatment, and then at 6 months, 1 year, 2 years, and 5 years after your treatment. The study staff may call you or ask you to come into the clinic to complete these questionnaires. It should take about 10-15 minutes to complete these questionnaires each time.
- Leftover tissue samples from biopsies or surgeries will be collected and stored in a research bank at MD Anderson for use in future research related to cancer.

Before your clinical information and tissue samples can be used for research, the researchers must get approval from the Institutional Review Board (IRB) of MD Anderson. The IRB is a committee of doctors, researchers, and community members. The IRB is responsible for protecting study participants and making sure all research is safe and ethical.

Your samples and data will be given a code number. No identifying information will be directly linked to your samples. Only the researcher in charge of the bank will have access to the code numbers and be able to link the samples to you. This is to allow medical data related to the samples to be updated as needed.

2. POSSIBLE RISKS

MD Anderson and others can learn about cancer and other diseases from your **banked tissue samples and data**. In the future, people who may do research with these samples may need to know more information about your health. This information may be collected from your medical record. MD Anderson will make reasonable efforts to preserve your privacy, but cannot guarantee complete privacy. Sometimes your samples may be used for genetic research about diseases that are passed on in families.

MD Anderson will not be able to give you, your family, or your doctor the reports about the research done with your data, and these reports will not be put in your health records. If this information were released to you, your family, or third parties, it could be misused. Such misuse could be distressing, and it could cause you or your family members to have difficulty obtaining insurance coverage and/or a job. In the future, people who may do research with your data may need to know more information about your health. This information may be collected from your medical record. If your data were used for this kind of research, the results would not be put in your health records.

Genetic research may result in the development of beneficial treatments, devices, new drugs, or patentable procedures. If this happens, there are no plans to compensate you. Specific genetic studies might be designed to gather information that could be used by your doctor to select therapy for you. In this case, this information would be given to your MD Anderson doctor to discuss with you.

If you withdraw your consent to the storage of data in the research database or leftover samples in the tissue bank, then they will no longer be collected for storage. Any of your data that remains in the research database or samples that remain in the tissue bank will no longer be used for research and will be destroyed.

However, if any of your de-identified samples or data were already released for research purposes before you withdrew consent, MD Anderson will not be able to destroy or delete it.

Questionnaires may contain questions that are sensitive in nature. You may refuse to answer any question that makes you feel uncomfortable. If you have concerns about completing the questionnaire, you are encouraged to contact your doctor or the study chair.

This research is covered by a **Certificate of Confidentiality (CoC)** from the National Institutes of Health. The researchers with this CoC may not disclose or use information that may identify you in any federal, state, or local civil, criminal, administrative, legislative, or other action, suit, or proceeding, or be used as evidence, for example, if there is a court subpoena, unless you have consented for this use. Information protected by this CoC cannot be disclosed to anyone else who is not connected with the research except, if there is a federal, state, or local law that requires disclosure (such as to report child abuse or communicable diseases but not for federal, state, or local civil, criminal, administrative, legislative, or other proceedings, see below).

The CoC cannot be used to refuse a request for information from personnel of the United States federal or state government agency sponsoring the project that is needed for auditing or program evaluation. You should understand that a CoC does not prevent you from voluntarily releasing information about yourself or your involvement in this research. If you want your research information released to an insurer, medical care provider, or any other person not connected with the research, you must provide consent to allow the researchers to release it.

The CoC will not be used to prevent disclosure for any purpose you have consented to.

Although every effort will be made to keep study data safe, there is a chance that your personal health information could be lost or stolen, which may result in a **loss of confidentiality**. All study data will be stored in password-protected computers and/or locked file cabinets and will continue to be stored securely after the study. Only authorized study staff will have access to study data.

This study may involve unpredictable risks to the participants.

MD Anderson IRB Approved 07/22/2024

OPTIONAL PROCEDURES FOR THE STUDY

You do not have to agree to the optional procedures in order to take part in this study. There are no benefits to you for taking part in the optional procedures. Future patients may benefit from what is learned. You may stop taking part at any time. There will be no cost to you for taking part in the optional procedures.

Optional Procedure #1: If you agree, you may complete up to 4 questionnaires about your quality of life and side effects from treatment 1 time every year from 6-10 years after your treatment. The study staff may call you or ask you to come into the clinic to complete these questionnaires. It should take about 10-15 minutes to complete these questionnaires each time.

This study may also want to collect information about your diet and nutrition. Researchers will use this information for use in future research related to cancer.

Optional Procedure #2: If you agree, you will have an electromyography (EMG) procedure on your head or neck to measure your muscle activity in those areas. You will have a lymphedema/fibrosis physical and functional assessment to assess hypoglossal nerve activity. Additionally, an electrical impedance myograph (EIM)/surface EMG (sEMG) with a user tongue array (UTA) depressor will be used to measure your tongue surface.

- During an EMG procedure, a sensor pad will be placed on your head or neck and a needle will also be placed for a few seconds in your skin on a muscle on your neck to measure muscle activity. If you agree, you will have this test performed either at pre-treatment, less than 12 months after radiation, or at least 12 months after radiation.

- During the EIM/sEMG with the UTA depressor, the depressor will be placed on your tongue. It will then transmit a weak, painless electric signal into your tongue to measure the electrical composition and structure of your tongue. This procedure will be implemented on all patients, irrespective of at which time point the participant is enrolled in the study.

Optional Procedure #3: If you agree, blood (about 4 teaspoons each time) will be drawn before treatment, 3 weeks after starting treatment, and at least one time during surveillance.

<u>Optional Procedure Risks:</u>

Questionnaires may contain questions that are sensitive in nature. You may refuse to answer any question that makes you feel uncomfortable. If you have concerns about completing the questionnaire, you are encouraged to contact your doctor or the study chair.

EMG may rarely cause bleeding, bruising, and/or muscle spasms.

MD Anderson IRB Approved 07/22/2024

EIM/sEMG with a UTA depressor may cause brief, minor tongue irritation due when the depressor is placed on the tongue. You may experience tingling in the tongue during the measurement. However, this is very unlikely. If you are experiencing discomfort, the depressor will be removed from your mouth.

<u>CONSENT/PERMISSION/AUTHORIZATION FOR OPTIONAL PROCEDURES</u>

Circle your choice of “yes” or “no” for the following optional procedures:

Optional Procedure #1: Do you agree to be complete up to 4 questionnaires about your quality of life and side effects from treatment annually from 6 to 10 years after your treatment?

YES **NO**

Optional Procedure #2: Do you agree to have your muscle activity measured using EMG, a lymphedema/fibrosis physical, and your tongue surface measured using EIM/sEMG with a UTA depressor at one of the timepoints described above?

YES **NO**

Optional Procedure #3: Do you agree to have blood drawn before treatment, 3 weeks after starting treatment, and at least one time during surveillance?

YES **NO**

3. COSTS AND COMPENSATION

If you suffer injury as a direct result of taking part in this study, MD Anderson health providers will provide medical care. However, this medical care will be billed to your insurance provider or you in the ordinary manner. You will not be reimbursed for expenses or compensated financially by MD Anderson for this injury. You may also contact the Chair of MD Anderson’s IRB at 713-792-6477 with questions about study-related injuries. By signing this consent form, you are not giving up any of your legal rights

Certain tests, procedures, and/or drugs that you may receive as part of this study may be without cost to you because they are for research purposes only. However, your insurance provider and/or you may be financially responsible for the cost of care and treatment of any complications resulting from the research tests, procedures, and/or drugs. Standard medical care that you receive under this research study will be billed to your insurance provider and/or you in the ordinary manner. Before taking part in this study, you may ask about which parts of the research-related care may be provided without charge, which costs your insurance provider may pay for, and which costs may be your responsibility. You may ask that a financial counselor be made available to you to talk about the costs of this study.

Samples that are collected from you in this study may be used for the development of treatments, devices, new drugs, or patentable procedures that may result in commercial profit.

There are no plans to compensate you for any patents or discoveries that may result from your participation in this research.

You will receive no compensation for taking part in this study.

Additional Information

4. You may ask the study chair (Dr. Amy Moreno, at 713-745-4590) any questions you have about this study. You may also contact the Chair of MD Anderson's Institutional Review Board (IRB - a committee that reviews research studies) at 713-792-6477 with any questions that have to do with this study or your rights as a study participant.

5. You may choose not to take part in this study without any penalty or loss of benefits to which you are otherwise entitled. You may also withdraw from participation in this study at any time without any penalty or loss of benefits. If you withdraw from this study, you can still choose to be treated at MD Anderson.

   If you stop being in the research, already collected data may not be removed from the study database. You may be asked whether the study doctor can continue to collect data from your routine medical care. If you agree, this data will be handled the same as research data.

6. This study or your participation in it may be changed or stopped at any time by the study chair, or the IRB of MD Anderson.

7. MD Anderson may benefit from your participation and/or what is learned in this study.

Future Research

Data

Your personal information is being collected as part of this study. These data may be used by researchers at MD Anderson and/or shared with other researchers and/or institutions for use in future research.

Samples

Samples (such as blood and/or tissue) are being collected from you as part of this study. Researchers at MD Anderson may use any leftover samples that is stored at MD Anderson in future research.

If identifiers are removed from your identifiable private information or identifiable samples that are collected during this research, that information or those samples

could be used for future research studies or shared with another researcher for future research studies without your additional informed consent.

In some cases, all of your identifying information may not be removed before your data or research samples are used for future research. If future research is performed at MD Anderson, the researchers must get approval from the Institutional Review Board (IRB) of MD Anderson before your data and/or research samples can be used. At that time, the IRB will decide whether or not further permission from you is required. The IRB is a committee of doctors, researchers, and community members that is responsible for protecting study participants and making sure all research is safe and ethical.

If you do not want your samples or data to be used for future research, tell the study doctor. You may withdraw your samples at any time by telling your study team. If you decide to withdraw your samples, they will be returned to the lab they came from or destroyed. However, the data and test results already collected from your samples will be kept and may be used.

If this research is not performed at MD Anderson, MD Anderson will not have oversight of any data and/or samples.

Genetic Research
Research samples collected from you as part of this study may/will be used for genetic research, which may include whole genome sequencing. Whole genome sequencing is a type of testing in which researchers study your entire genetic makeup (DNA). This may help researchers learn how changes in the ordering of genes may affect a disease or response to treatment. If genetic research is done with your samples, those who have access to those samples may be able to identify you. The results of this research may also be able to be linked to you.

A federal law, called the Genetic Information Nondiscrimination Act (GINA), generally makes it illegal for health insurance companies, group health plans, and most employers to discriminate against you based on your genetic information. This law generally will protect you in the following ways:

- Health insurance companies and group health plans may not request your genetic information that we get from this research.
- Health insurance companies and group health plans may not use your genetic information when making decisions regarding your eligibility or premiums.
- Employers with 15 or more employees may not use your genetic information that we get from this research when deciding to hire, promote, or fire you or when setting the terms of your employment.

Be aware that this federal law does not protect you against genetic discrimination by companies that sell life insurance, disability insurance, or long-term care insurance. Nor does this federal law prohibit discrimination based on an already known genetic disease or disorder.

Authorization for Use and Disclosure of Protected Health Information (PHI):

A. During the course of this study, MD Anderson will be collecting and using PHI, including identifying information, information from your medical record, and study results. For legal, ethical, research, and safety related reasons, your doctor and the research team may share your PHI with:

- Federal agencies that require reporting of clinical study data (such as the FDA, National Cancer Institute [NCI], and OHRP)
- The IRB and officials of MD Anderson
- Study monitors and auditors who verify the accuracy of the information
- Individuals who put all the study information together in report form

Study sponsors and/or supporters receive limited amounts of PHI. They may also view additional PHI in study records during the monitoring process. MD Anderson's contracts require sponsors/supporters to protect this information and limit how they may use it.

To protect your identity, the samples collected from you will be labeled with a unique number instead of your name or other identifying information. Only the study doctor or study staff will have access to the code that can link you to your samples.

The data collected from the user tongue array (UTA) depressor in the patients who participate in the EMG study will be de-identified and sent to Dr. Benjamin Sanchez at the University of Utah for analysis.

The results of this research may be published in scientific journals or presented at medical meetings, but your identity will not be disclosed.

B. Signing this consent and authorization form is optional but you cannot take part in this study if you do not agree and sign.

C. MD Anderson will do its best to protect the privacy of your records, but it is possible that once information is shared with people listed on this form, it may be released to others. If this happens, your information may no longer be protected by federal law.

D. The permission to use your PHI will continue indefinitely unless you withdraw your authorization in writing. Instructions on how to do this can be found in the MD Anderson Notice of Privacy Practices (NPP) or you may contact the Chief Privacy Officer of MD Anderson at 713-745-6636. If you withdraw your authorization, you will be removed from the study and the data collected about you up to that point can be used and included in data analysis. However, no further information about you will be collected.

CONSENT/AUTHORIZATION

I understand the information in this consent form. I have had a chance to read the consent form for this study, or have had it read to me. I have had a chance to think about it, ask questions, and talk about it with others as needed. I give the study chair permission to enroll me on this study. By signing this consent form, I am not giving up any of my legal rights. I will be given a signed copy of this consent document.

_______________________________________ ________
SIGNATURE OF PARTICIPANT DATE

____
PRINTED NAME OF PARTICIPANT

WITNESS TO CONSENT

I was present during the explanation of the research to be performed under this protocol.

____ _________
SIGNATURE OF WITNESS TO THE VERBAL CONSENT DATE
PRESENTATION (OTHER THAN PHYSICIAN OR STUDY CHAIR)
A witness signature is only required for non-English speakers utilizing the short form consent process (VTPS) and patients who are illiterate.

____
PRINTED NAME OF WITNESS TO THE VERBAL CONSENT

PERSON OBTAINING CONSENT

I have discussed this research study with the participant and/or his or her authorized representative, using language that is understandable and appropriate. I believe that I have fully informed this participant of the nature of this study and its possible benefits and risks and that the participant understood this explanation.

____ _________
PERSON OBTAINING CONSENT DATE

____
PRINTED NAME OF PERSON OBTAINING CONSENT

TRANSLATOR

I have translated the above informed consent as written (without additions or subtractions) into______________________________and assisted the people
(Name of Language)
obtaining and providing consent by translating all questions and responses during the consent process for this participant.

____________________ ______________________________ ___________
NAME OF TRANSLATOR SIGNATURE OF TRANSLATOR DATE

☐ Please check here if the translator was a member of the research team. (If checked, a witness, other than the translator, must sign the witness line.)

MD Anderson IRB Approved 07/22/2024
