# Supplementary material for: A cross-sectional protocol for experimental tongue high-density surface electromyography to detect and classify radiation-associated hypoglossal neuropathy
Source: PLoS One. 2026 Apr 29;21(4):e0347891. doi: 10.1371/journal.pone.0347891 (PMC13127915; doi:10.1371/journal.pone.0347891)
Supplement: S2 File — This is the Spanish parent consent form for #PA14–0947. See pages 4–6 for the optional procedures #2 consent. (PDF) [file pone.0347891.s002.pdf]

THE UNIVERSITY OF TEXAS
MD Anderson ~~Cancer~~ Center

Consentimiento informado

CONSENTIMIENTO INFORMADO/AUTORIZACIÓN PARA PARTICIPAR EN UNA INVESTIGACIÓN CON PROCEDIMIENTOS OPCIONALES

Base de datos del programa de orofaringe
(Oropharynx Program Database)
PA14-0947

Investigadora principal: Amy Moreno, MD

________________________________ ________________________
Nombre del/de la participante Número de expediente médico

Este documento es un formulario de consentimiento informado y autorización para un estudio de investigación, e incluye un resumen del estudio. Después del resumen, se brinda una descripción más detallada de los procedimientos y riesgos.

Esta investigación ha sido revisada y aprobada por una Institutional Review Board (Junta de Revisión Institucional, o IRB, por sus siglas en inglés; un comité que revisa los estudios de investigación).

RESUMEN DEL ESTUDIO

El objetivo de este estudio de investigación es recopilar información y muestras de tejido tumoral sobrantes de pacientes con cáncer de cabeza y cuello, como de orofaringe, amígdalas y la base de la lengua. Los investigadores usarán estas muestras e información para crear una base de datos y un banco de investigación con el fin de usarlos en investigaciones futuras relacionadas con el cáncer.

Este es un estudio de investigación.

Los futuros pacientes podrían beneficiarse de lo que se aprenda en este estudio.
No hay beneficios para usted en este estudio.

Su participación es totalmente voluntaria. Antes de elegir participar en este estudio, debe hablar con el equipo del estudio sobre las dudas que tenga, incluidos los posibles gastos y compromiso de tiempo.

MD Anderson IRB Approved 08/01/2024

A continuación, en la sección “Posibles Riesgos” del presente consentimiento, encontrará una lista de los posibles riesgos.

No habrá ningún costo para usted por participar en este estudio.

Usted puede elegir no participar en este estudio.

1. DETALLES DEL ESTUDIO

En este estudio participarán hasta 5000 pacientes, todos ellos, en MD Anderson.

Si usted acepta participar en este estudio:

- El personal del estudio recopilará información sobre su enfermedad y su historial de tratamientos de su expediente médico.
- Es posible que le pidan que responda hasta 4 cuestionarios sobre su calidad de vida y los efectos secundarios del tratamiento antes de su tratamiento programado de la forma habitual, durante el tratamiento y, luego, a los 6 meses, al año, a los dos años y a los 5 años después del tratamiento. Es posible que el personal del estudio lo/la llame o le pida que vaya a la clínica para responder estos cuestionarios. Contestar estos cuestionarios le tomará alrededor de 10-15 minutos en cada ocasión.
- Se obtendrán las muestras de tejido sobrantes de las biopsias o cirugías y se almacenarán en un banco de investigación en MD Anderson para su uso en futuras investigaciones relacionadas con el cáncer.

Antes de poder utilizar su información clínica y sus muestras de tejidos con fines de investigación, los investigadores deberán obtener una aprobación de la Institutional Review Board (IRB) de MD Anderson. La IRB es un comité formado por médicos, investigadores y miembros de la comunidad y es responsable de proteger a los participantes de los estudios y de garantizar que toda investigación se lleve a cabo de manera segura y ética.

Sus muestras y datos recibirán un código numérico. Ninguna información que pueda identificarlo/a estará relacionada directamente con sus muestras. Solo el/la investigador/a a cargo del banco tendrá acceso a los códigos numéricos y podrá vincular las muestras con usted. Esto tiene como objetivo permitir que los datos médicos relacionados con las muestras puedan actualizarse cuando sea necesario.

2. POSIBLES RIESGOS

MD Anderson y otros pueden aprender acerca del cáncer y otras enfermedades a partir de sus **muestras de tejido y datos almacenados**. Es posible que en el futuro quienes realicen alguna investigación con estas muestras necesiten saber más acerca de su salud. Esta información se podría obtener de su expediente médico.
MD Anderson hará todo lo razonablemente posible por mantener su privacidad, pero no puede garantizarla por completo. En ocasiones, sus muestras podrían utilizarse para investigaciones genéticas sobre enfermedades hereditarias.

MD Anderson IRB Approved 08/01/2024

MD Anderson no le entregará a usted, ni a su familia ni a su médico/a los informes de la investigación realizada con estos datos, y dichos informes tampoco aparecerán en su expediente médico. Si esta información se la proporcionaran a usted, a su familia o a otras personas, podría ser utilizada indebidamente. Dicho uso indebido podría ser causa de angustia y hacer que usted o sus familiares tuvieran dificultades para obtener una cobertura de seguro o un empleo. Es posible que, en el futuro, quienes hagan investigaciones con sus datos necesiten saber más acerca de su salud. Esta información se podría obtener de su expediente médico. Si su información se utilizara para este tipo de investigación, los resultados no se incluirían en su expediente médico.

Las investigaciones genéticas pueden dar lugar al desarrollo de tratamientos beneficiosos, dispositivos, medicamentos nuevos o procedimientos patentables.
No existen planes para ofrecerle ninguna compensación en caso de que esto ocurra. Se podrían diseñar estudios genéticos específicos a fin de recopilar información que su médico/a podría usar para seleccionar el tratamiento para usted. De ser así, esta información se entregará a su médico/a de MD Anderson para que hable de ella con usted.

Si usted retira su consentimiento para el almacenamiento de datos en la base de datos de la investigación o de las muestras sobrantes en el banco de tejidos, estos ya no se obtendrán para su almacenamiento. Todos sus datos que permanezcan en la base de datos de la investigación y sus muestras que se conserven en el banco de tejidos dejarán de utilizarse para fines de investigación y se destruirán.

Sin embargo, si alguna de sus muestras o de sus datos sin identificación ya se hubiera compartido con fines de investigación antes de que usted retirara su consentimiento, MD Anderson no podrá destruirlos ni eliminarlos.

Los **cuestionarios** pueden incluir preguntas de naturaleza delicada. Usted puede negarse a responder cualquier pregunta que le haga sentir incomodidad. Si tiene alguna pregunta acerca de cómo contestar el cuestionario, le aconsejamos que contacte a su médico/a o a la investigadora principal.

Esta investigación está cubierta por un ***Certificado de confidencialidad*** (CoC, por sus siglas en inglés) de los National Institutes of Health (Institutos Nacionales de Salud). Los investigadores amparados por este *Certificado de confidencialidad* no pueden divulgar o usar la información que pueda identificarlo/a en ninguna demanda, juicio o procedimiento federal, estatal o local, ya sea civil, penal, administrativo, legislativo o de otra índole, ni pueden utilizar dicha información como prueba, por ejemplo, en caso de citación judicial, a menos que usted haya firmado un consentimiento autorizando dicho uso. La información protegida por este *Certificado de confidencialidad* no puede divulgarse a nadie que no tenga relación con la investigación, salvo que haya una ley federal, estatal o local que requiera la divulgación (como la legislación que obliga a denunciar el abuso infantil o las enfermedades transmisibles, pero no para procesos federales, estatales o locales, ya

sean civiles, penales, administrativos, legislativos o de otra índole). (Para más información, véase a continuación).

El *Certificado de confidencialidad* no puede usarse para rechazar una solicitud de información emitida por personal de organismos gubernamentales federales o estatales de los Estados Unidos que patrocinan el proyecto cuando se solicite con fines de auditoría o evaluación del programa. Debe entender que el *Certificado de confidencialidad* no impide que usted voluntariamente revele información sobre sí mismo/a o sobre su participación en esta investigación. Si desea que su información de la investigación se comparta con una compañía aseguradora, un proveedor de atención médica o cualquier otra persona que no tenga relación con la investigación, debe dar su consentimiento para que los investigadores puedan compartirla.

El *Certificado de confidencialidad* no se utilizará para evitar ninguna divulgación para la que usted haya dado su consentimiento.

Aunque se tomarán todas las medidas posibles para mantener la seguridad de los datos del estudio, existe la posibilidad de que su información de salud personal se pierda o sea robada, lo que puede dar lugar a una **pérdida de confidencialidad**. Todos los datos del estudio se almacenarán en computadoras protegidas por contraseña o armarios cerrados con llave, y permanecerán almacenados de manera segura después del estudio. Solo el personal autorizado del estudio tendrá acceso a los datos del estudio.

Este estudio puede implicar riesgos impredecibles para los participantes.

PROCEDIMIENTOS OPCIONALES DEL ESTUDIO

Para participar en este estudio usted no tiene la obligación de participar en los procedimientos opcionales. No hay beneficios para usted por participar en los procedimientos opcionales. Los futuros pacientes podrían beneficiarse de lo que se aprenda. Usted puede dejar de participar en cualquier momento. No habrá costos para usted por participar en los procedimientos opcionales.

Procedimiento opcional n.º 1: Si está de acuerdo, es posible que responda hasta 4 cuestionarios sobre su calidad de vida y los efectos secundarios del tratamiento una (1) vez al año de 6 a 10 años después de su tratamiento. Es posible que el personal del estudio lo/la llame o le pida que vaya a la clínica para responder estos cuestionarios. Contestar estos cuestionarios le tomará alrededor de 10-15 minutos en cada ocasión.

Es posible que en este estudio también se quiera recopilar información sobre su dieta y nutrición. Los investigadores utilizarán esta información para investigaciones futuras relacionadas con el cáncer.

Procedimiento opcional n.º 2: Si está de acuerdo, le harán una electromiografía (EMG) en la cabeza o el cuello para medir su actividad muscular en esas áreas.

Le harán una evaluación física y funcional del linfedema/fibrosis para evaluar la actividad del nervio hipogloso. Además, se utilizará un miógrafo de impedancia eléctrica (EIM, por sus siglas en inglés) o una EMG de superficie [sEMG, por sus siglas en inglés]) con un depresor lingual para ensayo de usuario (UTA, por sus siglas en inglés) para medir la superficie de la lengua.

- Durante una EMG, le colocarán una almohadilla con un sensor en la cabeza o el cuello y también le introducirán una aguja durante unos segundos en la piel, en un músculo del cuello, para medir la actividad muscular. Si está de acuerdo, le harán esta prueba antes del tratamiento, menos de 12 meses después de la radioterapia, o al menos 12 meses después de radioterapia.

- Durante el EIM o la sEMG con el depresor UTA, le colocarán el depresor en la lengua. A continuación, el dispositivo transmitirá una señal eléctrica débil que no produce dolor a la lengua para medir su composición eléctrica y su estructura. Este procedimiento se aplicará a todos los pacientes, independientemente del momento en que los participantes se inscriban en el estudio.

Procedimiento opcional n.º 3: si está de acuerdo, le extraerán sangre (unas 4 cucharaditas cada vez) antes del tratamiento, 3 semanas después de iniciarlo y al menos una vez durante la vigilancia.

Riesgos de los procedimientos opcionales:

Los **cuestionarios** pueden incluir preguntas de naturaleza delicada. Usted puede negarse a responder cualquier pregunta que le haga sentir incomodidad. Si tiene alguna pregunta acerca de cómo contestar el cuestionario, le aconsejamos que contacte a su médico/a o a la investigadora principal.

En raras ocasiones, la **EMG** puede provocar sangrado, moretones o espasmos musculares.

El EIM o la sEMG con un depresor UTA puede causar una irritación leve y pasajera de la lengua cuando se coloca el depresor en la lengua. Es posible que sienta un hormigueo en la lengua durante la medición, aunque es muy poco probable. Si siente molestias, le retirarán el depresor de la boca.

MD Anderson IRB Approved 08/01/2024

CONSENTIMIENTO/PERMISO/AUTORIZACIÓN PARA LOS PROCEDIMIENTOS OPCIONALES

Marque con un círculo "Sí" o "No" para indicar su respuesta a los siguientes procedimientos opcionales:

Procedimiento opcional n.º 1: ¿Está de acuerdo en rellenar hasta 4 cuestionarios sobre su calidad de vida y los efectos secundarios del tratamiento una vez al año, de 6 a 10 años después de su tratamiento?

SÍ **NO**

Procedimiento opcional n.º 2: ¿Está de acuerdo en que le midan la actividad muscular mediante EMG, que le hagan un examen físico de linfedema/fibrosis y que le midan la superficie de la lengua mediante EIM/sEMG con un depresor UTA en uno de los momentos descritos anteriormente?

SÍ **NO**

Procedimiento opcional n.º 3: ¿Está de acuerdo en que le extraigan sangre antes del tratamiento, 3 semanas después de iniciarlo y al menos una vez durante la vigilancia?

SÍ **NO**

3. COSTOS Y COMPENSACIÓN

Si usted sufre alguna lesión como resultado directo de su participación en este estudio, los profesionales de la salud de MD Anderson le brindarán atención médica. Sin embargo, esa atención médica se facturará a su seguro médico o a usted de la forma habitual. MD Anderson no le reembolsará los gastos ni le ofrecerá compensación económica por dicha lesión. También puede comunicarse con el/la presidente de la IRB de MD Anderson llamando al 713-792-6477 si tiene preguntas sobre las lesiones relacionadas con el estudio. Al firmar este formulario de consentimiento, usted no renuncia a ninguno de sus derechos legales.

Es posible que ciertas pruebas, procedimientos y/o medicamentos que usted pueda recibir como parte de este estudio no tengan ningún costo para usted, dado que se utilizan únicamente con fines de investigación. Sin embargo, su seguro médico y/o usted podrían ser económicamente responsables del costo de la atención médica y del tratamiento de las posibles complicaciones que resulten de las pruebas, los procedimientos o los medicamentos de la investigación. La atención médica estándar que reciba mientras participe en este estudio de investigación se facturará a su seguro médico y/o a usted de la manera habitual. Antes de participar en este estudio, puede preguntar qué partes de la atención relacionada con la investigación podría recibir sin cargos, qué costos podría pagar su seguro médico y cuáles podrían ser su responsabilidad. Puede pedir que le pongan a su disposición un/a asesor/a de finanzas para hablar sobre los costos de este estudio.

MD Anderson IRB Approved 08/01/2024

Las muestras que se obtengan de usted en este estudio pueden usarse para el desarrollo de tratamientos, dispositivos, nuevos medicamentos o procedimientos patentables que pueden generar beneficios comerciales.

No hay ningún plan para compensarlo/a por ninguna patente ni descubrimiento que pueda resultar de su participación en esta investigación.

Usted no recibirá ninguna compensación por participar en este estudio.

Información adicional

4. Puede comunicarse con la investigadora principal, la Dra. Amy Moreno, llamando al 713-745-4590, para cualquier pregunta que tenga acerca de este estudio. También puede comunicarse con el/la presidente de la Institutional Review Board (IRB, un comité que revisa los estudios de investigación) de MD Anderson llamando al 713-792-6477 para cualquier pregunta relacionada con este estudio o con sus derechos como participante.

5. Usted puede elegir no participar en este estudio sin ninguna sanción ni pérdida de los beneficios a los que tiene derecho. También puede retirarse de este estudio en cualquier momento sin ninguna sanción ni pérdida de beneficios. Si se retira de este estudio, aún puede elegir recibir tratamiento en MD Anderson.

   Si deja de participar en la investigación, los datos ya recopilados no se podrán eliminar de la base de datos del estudio. Es posible que le pregunten si la médica del estudio puede continuar recopilando datos de su atención médica de rutina. Si está de acuerdo, estos datos se manejarán igual que los de la investigación.

6. La investigadora principal o la IRB de MD Anderson pueden cambiar o suspender este estudio o su participación en el mismo en cualquier momento.

7. MD Anderson podría beneficiarse de su participación o de lo que se aprenda en este estudio.

Investigaciones futuras

Datos

Como parte de este estudio, se obtiene su información personal. Los investigadores de MD Anderson podrán utilizar esos datos o compartirlos con otros investigadores y/o instituciones para su uso en investigaciones futuras.

Muestras

Como parte de este estudio, le tomarán muestras (por ejemplo, de sangre y/o tejidos). Los investigadores de MD Anderson podrán usar las muestras sobrantes que se almacenen en MD Anderson en investigaciones futuras.

Si se eliminan los identificadores de su información privada o de las muestras que se obtienen en esta investigación, esa información o esas muestras podrían usarse en estudios de investigación futuros o compartirse con otros investigadores para estudios futuros de investigación sin su consentimiento informado adicional.

En algunos casos, es posible que no se elimine toda su información de identificación antes de que sus datos o muestras se usen en investigaciones futuras. Si la investigación futura se realiza en MD Anderson, los investigadores deberán obtener la aprobación de la Institutional Review Board (IRB) de MD Anderson antes de que se puedan usar sus datos y/o muestras. En ese momento, la IRB decidirá si es necesario o no obtener un permiso adicional suyo. La IRB es un comité de médicos, investigadores y miembros de la comunidad que es responsable de proteger a los participantes de los estudios y asegurarse de que todas las investigaciones sean seguras y éticas.

Si no desea que sus muestras o datos se utilicen en investigaciones futuras, comuníqueselo a la médica del estudio. Usted puede retirar sus muestras en cualquier momento comunicándoselo al equipo del estudio. Si decide retirar sus muestras, estas se devolverán al laboratorio del que proceden o se destruirán. Sin embargo, los datos y los resultados de las pruebas ya obtenidos de sus muestras se conservarán y podrán utilizarse.

Si la investigación en cuestión no se realiza en MD Anderson, MD Anderson no tendrá poder de supervisión sobre ningún dato ni muestra.

Investigaciones genéticas
Las muestras para investigación tomadas de usted como parte de este estudio podrán usarse/se usarán para investigaciones genéticas, que podrán incluir secuenciación del genoma completo. La secuenciación del genoma completo es un tipo de prueba en la que los investigadores estudian toda la composición genética (ADN) de la persona. Esto puede ayudar a los investigadores a aprender cómo los cambios del orden de los genes pueden afectar a una enfermedad o la respuesta al tratamiento. Si se realiza una investigación genética con sus muestras, es posible que quienes accedan a esas muestras puedan identificarlo/a. También es posible que los resultados de la investigación se puedan vincular con usted.

Una ley federal, llamada Ley de No Discriminación por Información Genética (GINA, por sus siglas en inglés), establece que, por lo general, es ilegal la discriminación contra una persona basada en su información genética por parte de compañías de seguros de salud, planes de salud grupales y la mayoría de los empleadores. Generalmente, esta ley protege de las siguientes maneras:

- Las compañías de seguros y los planes de salud grupales no podrán solicitar la información genética que hayamos obtenido de usted a través de esta investigación.
- Las compañías de seguros y los planes de salud grupales no podrán utilizar su información genética para tomar decisiones sobre su elegibilidad o primas.

MD Anderson IRB Approved 08/01/2024

- Los empleadores con 15 o más empleados no podrán utilizar su información genética obtenida en esta investigación para tomar decisiones de contratación, promoción o despido ni al establecer los términos de su empleo.

Tenga en cuenta que esta ley federal no protege contra la discriminación genética por parte de las compañías que venden seguros de vida, de discapacidad o de atención médica a largo plazo. Esta ley federal tampoco prohíbe la discriminación por una enfermedad o trastorno genético ya conocido.

Autorización para el uso y la divulgación de la información de salud protegida (PHI, por sus siglas en inglés):

A. Durante el transcurso de este estudio, MD Anderson recopilará y usará información de salud protegida (PHI), incluida la información que pueda identificarlo/a, información de su expediente médico y los resultados del estudio. Por motivos legales, éticos, de investigación y relacionados con la seguridad, su médica y el equipo de investigación pueden compartir su PHI con:
   - Las agencias federales que requieran la presentación de los datos de los estudios clínicos (como la Food and Drug Administration [Administración de Alimentos y Medicamentos, o FDA, por sus siglas en inglés]), el National Cancer Institute [Instituto Nacional del Cáncer, o NCI, por sus siglas en inglés] y la Office for Human Research Protections [Oficina para la Protección de Sujetos Humanos en Estudios de Investigación, u OHRP, por sus siglas en inglés])
   - La IRB y los funcionarios de MD Anderson
   - Los monitores y los auditores del estudio que verifiquen la fidelidad de la información
   - Las personas que recopilen toda la información del estudio en informes

   Los patrocinadores o auspiciantes el estudio reciben cantidades limitadas de PHI. También podrán ver partes adicionales de PHI en los expedientes del estudio durante el proceso de monitoreo. Los contratos de MD Anderson requieren que los patrocinadores o auspiciantes protejan esta información, y limitan la forma en que la pueden utilizar.

   Para proteger su identidad, las muestras que se obtengan de usted se etiquetarán con un número único en lugar de su nombre u otra información de identificación. Solo la médica del estudio o el personal del estudio tendrán acceso al código que puede vincularlo/a a sus muestras.

   Los datos recopilados del depresor lingual para ensayo de usuario (UTA) en los pacientes que participen en la EMG se anonimizarán y se enviarán al Dr. Benjamin Sanchez de University of Utah para su análisis.

   Los resultados de esta investigación se podrán publicar en revistas científicas o presentar en conferencias médicas, pero no se revelará su identidad.

MD Anderson IRB Approved 08/01/2024

B. Firmar este formulario de consentimiento y autorización es opcional, pero usted no podrá participar en este estudio si no está de acuerdo y no lo firma.

C. MD Anderson hará todo lo posible para proteger la privacidad de sus expedientes, pero es posible que, una vez que la información se comparta con las personas que figuran en este formulario, se divulgue a terceros. Si esto ocurre, es posible que su información deje de estar protegida por la ley federal.

D. El permiso para usar su PHI continuará vigente por tiempo indefinido, a menos que usted retire su autorización por escrito. Encontrará las instrucciones sobre cómo hacerlo en el *Aviso de prácticas de privacidad* (NPP, por sus siglas en inglés) de MD Anderson. También puede comunicarse con el/la director/a de privacidad de MD Anderson llamando al 713-745-6636. Si retira su autorización, lo/la retirarán del estudio y los datos recopilados sobre usted hasta ese momento podrán utilizarse e incluirse en el análisis de datos. Sin embargo, no se obtendrá información adicional sobre usted.

MD Anderson IRB Approved 08/01/2024

CONSENTIMIENTO/AUTORIZACIÓN

Entiendo la información de este formulario de consentimiento. He tenido la oportunidad de leer el formulario de consentimiento de este estudio, o de que alguien me lo leyera. Asimismo, he tenido la oportunidad de pensar al respecto, hacer preguntas y hablar con otras personas en la medida de lo necesario. Doy mi permiso a la investigadora principal para que me inscriba en este estudio. Al firmar este formulario de consentimiento no renuncio a ninguno de mis derechos legales. Me darán una copia de este documento de consentimiento firmado.

____ _______________
FIRMA DEL/DE LA PARTICIPANTE FECHA

____
NOMBRE DEL/DE LA PARTICIPANTE
EN LETRA DE IMPRENTA

TESTIGO DEL CONSENTIMIENTO

Estuve presente durante la explicación de la investigación que se realizará bajo este protocolo.

____ _______________
FIRMA DEL/DE LA TESTIGO DE LA PRESENTACIÓN VERBAL DEL CONSENTIMIENTO (QUE NO SEA EL/LA MÉDICO/A NI LA INVESTIGADORA PRINCIPAL) FECHA

La firma de un/a testigo solo es necesaria si la persona no habla inglés y utiliza el formulario de consentimiento abreviado (VTPS), o si no sabe leer ni escribir.

____
NOMBRE DEL/DE LA TESTIGO DEL CONSENTIMIENTO
VERBAL, EN LETRA DE IMPRENTA

PERSONA QUE OBTIENE EL CONSENTIMIENTO

He hablado sobre este estudio de investigación con el/la participante o su representante autorizado/a, utilizando un lenguaje comprensible y apropiado. Considero que he informado completamente al/a la participante acerca de la naturaleza de este estudio, así como de sus posibles beneficios y riesgos, y que el/la participante comprendió esta explicación.

____ _______________
PERSONA QUE OBTIENE EL CONSENTIMIENTO FECHA

____
NOMBRE DE LA PERSONA QUE OBTIENE EL
CONSENTIMIENTO EN LETRA DE IMPRENTA

MD Anderson IRB Approved 08/01/2024

TRADUCTOR/A

He traducido el consentimiento informado anterior tal y como está escrito (sin adiciones ni omisiones) al____y he asistido a quienes
(Idioma)
obtenían y otorgaban el consentimiento traduciendo todas las preguntas y respuestas durante el procedimiento de consentimiento de este/a participante.

NOMBRE DEL/DE LA TRADUCTOR/A	FIRMA DEL/DE LA TRADUCTOR/A	FECHA

☐ Indique si el/la traductor/a era un miembro del equipo de investigación.
(De ser así, un/a testigo, que no sea el/la traductor/a, debe firmar en la línea de testigo).

MD Anderson IRB Approved 08/01/2024
